# The characteristics of auditorial event-related potential under propofol sedation associated with preoperative cognitive performance in glioma patients

**DOI:** 10.3389/fnins.2024.1431406

**Published:** 2024-11-14

**Authors:** Xinxin Wang, Wanning Yang, Minyu Jian, Yi Liang, Zuocheng Yang, Yiwei Chen, Bo Ma, Chengwei Wang, Zonggang Hou, Zhenghai Deng, Haiyang Liu, Jian Xie, Ruquan Han

**Affiliations:** ^1^Department of Anesthesiology, Beijing Tiantan Hospital, Capital Medical University, Beijing, China; ^2^Department of Neurosurgery, Beijing Tiantan Hospital, Capital Medical University, Beijing, China

**Keywords:** propofol sedation, mild cognitive impairment, auditory event-related potentials, theta-ERSP, glioma

## Abstract

**Background:**

Glioma patients often experience neurocognitive deficits, particularly mild cognitive impairment (MCI), which affects their perioperative safety. The use of auditory event-related potentials (AERPs) might be a promising method for reflecting perioperative cognitive function in patients, even under unresponsive sedation. In this study, we aimed to investigate the relationships between the AERP under sedation and preoperative cognitive performance in glioma patients.

**Methods:**

Patients with primary supratentorial gliomas who were scheduled for elective craniotomy under general anesthesia were included in this prospective observational study. The patients were categorized into MCI and non-MCI groups based on their preoperative Montreal Cognitive Assessment (MoCA) scores. AERP characteristics, including mismatch negativity (MMN), P300, and event-related spectral perturbation (ERSP) in the theta bands, were analyzed under different propofol-induced sedation conditions. Differences in these parameters between groups and their relationships with preoperative cognitive performance were subsequently investigated.

**Results:**

Twenty-nine eligible patients were included in the analysis. Compared to that in the non-MCI group, the average amplitude of the MMN component evoked by the novel stimulus significantly decreased during the recovery period in the MCI group (−3.895 ± 1.961 μV vs. -1.617 ± 1.831 μV, *p* = 0.003). Theta-ERSPs also differed between the two groups under standard (0.021 ± 0.658 μV^2^/Hz vs. 0.515 ± 0.622 μV^2^/Hz, *p* = 0.048) and novel (0.212 ± 0.584 μV^2^/Hz vs. 0.823 ± 0.931 μV^2^/Hz, *p* = 0.041) stimulation conditions under light sedation. After correcting for age, education level, site of lesion, WHO pathological grade and combined symptomatic epilepsy as confounders, the frontal theta-ERSP induced by standard and novel stimuli under light sedation was inversely related to the preoperative MoCA score (standard stimuli: *β* = −0.491, *p* = 0.011; novel stimuli: *β* = −0.594, *p* = 0.007), as was the average MMN amplitude induced by novel stimuli during the recovery period (β = −0.356, *p* = 0.035).

**Conclusion:**

The AERP neural response characteristics of glioma patients during propofol sedation were associated with preoperative cognitive performance, which might be a potential neurophysiological indicator for monitoring perioperative cognitive function, especially theta-ERSP.

## 1 Introduction

Glioma is one of the most common primary intracranial diseases and frequently presents with neurological deficits and cognitive impairments (CIs) (Schiff et al., 2019). Prior studies have demonstrated that neurocognitive performance is an essential prognostic factor of survival rates and a reliable predictor of tumor progression (Butterbrod et al., 2019; Noll et al., 2015). Therefore, perioperative cognitive monitoring and evaluation is necessary for both oncological therapy and clinical care. Evidence from former research suggested that preoperative CI posed a risk for adverse postoperative mental status changes and increased incidence of agitation during recovery from anesthesia (Fields et al., 2018). Both the type of anesthesia and the depth of anesthesia may affect the cognitive level of patients, especially during neuropathological states (Miller et al., 2018; Teng et al., 2022; Evered et al., 2021). Thus, perioperative identification and evaluation of CI could provide informative guidance for perioperative anesthesia management to avoid further damage to patients’ fragile cognitive abilities. However, there are no reliable indicators to characterize cognitive status under sedation, and it is still lacking neurophysiological markers for perioperative cognitive function monitoring.

Mild cognitive impairment (MCI) is defined as cognitive impairment that is of insufficient severity to constitute functional impairment. The assessment and diagnosis of MCI relies mainly on neuropsychological assessments. The Montreal Cognitive Assessment (MoCA) is one of the most commonly used scales for identifying MCI (Wang et al., 2023). Although the MoCA and other scales were initially designed for patients with MCI, these scales are still susceptible to the subjective factors of the tester, as well as the age and education context of patients. Furthermore, test scores do not provide information on test specificity or test sensitivity for detecting more subtle cognitive changes, nor can they be used to detect cognitive function under sedation.

Event-related potentials (ERPs) are strongly linked with endogenous cognitive activities. Consequently, ERPs are extensively employed in studies of cognitive function as an objective method to reflect advanced brain activity related to intellectual activities. Among the ERPs evoked by other sensory stimuli, auditory ERPs (AERPs) have been widely used in studies assessing brain information processing capacity under different states of sedation, given that they are relatively less affected by the state of consciousness (Koelsch et al., 2006). Mismatch negativity (MMN) and P300 are the most common AERP components; these components can detect subclinical cognitive decline in patients with normal behavior when performing certain cognitive tasks and provide objective, quantitative, neuro-electrophysiological markers (Doi et al., 2007).

Event-related spectral perturbations (ERSPs) are another method for capturing neural oscillation and can reflect task-related neuromodulation across different frequency bands. Previous studies have suggested that the theta band, especially theta activity in the frontal cortex, is significantly associated with cognitive workload (Chikhi et al., 2022). Thus, we speculate that frontal theta-ERSPs might be another candidate index for monitoring perioperative neurocognitive function. Yet the relationship between AERP characteristics and cognitive function under sedation remains unclear.

In this prospective pilot study, we aimed to investigate the relationships between the characteristics of the AERP in glioma patients under different propofol-induced sedation states and preoperative cognitive performance to better understand cognition-related neural responses in neuropathological states during sedation. We hypothesized that the characteristics of AERP neural responses in glioma patients under different propofol sedation states were associated with preoperative cognitive performance.

## 2 Materials and methods

### 2.1 Clinical trial registration and ethics statement

The prospective observational study was performed at Beijing Tiantan Hospital, Capital Medical University, from June 2022 to September 2023 and complied with the principles of the Declaration of Helsinki. This study was approved by the Chinese Ethics Committee of Registering Clinical Trials (ChiECRCT20220131,05/01/2022), and written informed consent was obtained from all participants. The study was registered prior to patient enrollment at ClinicalTrials.gov (NCT05352685; date of registration: 04/18/2022).

### 2.2 Participants

Patients with primary supratentorial gliomas who were scheduled for selective craniotomy under general anesthesia were included. The inclusion criteria for the study included ① aged 18–60 years, ② American Society of Anesthesiology status (ASA) I or II, ③ BMI ≤ 30 kg/m^2^, ④ native Chinese speaker, and ⑤ right-handed. The exclusion criteria were as follows: ① Mallampati airway classification ≥ III; ② hearing impairment; ③ recurrent or multiple intracranial tumors; ④ combined other psychoneurological disorders; and ⑤ a history of long-term smoking, drinking, or drug dependence.

Patients were categorized into the MCI group or the non-MCI group based on their preoperative neurocognitive performance. Cognitive function was assessed preoperatively using the MoCA. MCI status was determined using specific education cutoff points for total MoCA scores. According to Chinese MoCA norms, MoCA scores ≤13 indicate illiterate individuals, ≤19 indicates participants with primary school education, and ≤ 24 indicates those with middle school education and above. The total MoCA score (if less than 30) increased by 1 point when the respondent had no more than 12 years of education (Jia et al., 2021; Lu et al., 2011).

### 2.3 Propofol sedation protocol

The subjects laid comfortably on the bed after putting on the EEG cap and headphones. During the experiment, the participants were instructed to close their eyes and relax. Basic vital signs (pulse oximetry, blood pressure, electroencephalography, and temperature) were monitored throughout the experiment.

**Figure 1:**
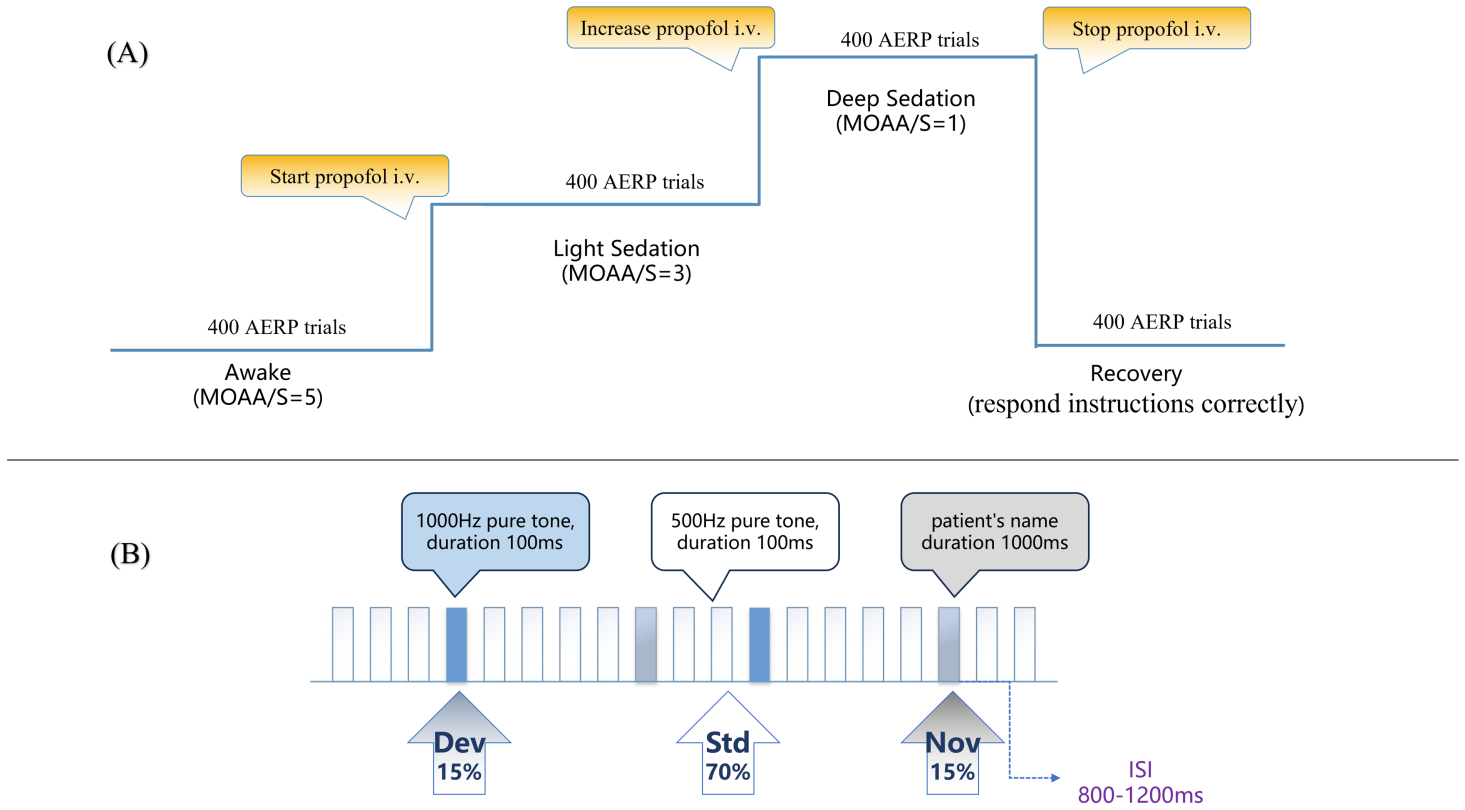
Study flow diagram and oddball auditory stimuli paradigm. **(A)** Study flow diagram. **(B)** Oddball auditory stimuli paradigm. AERP: auditory event-related potential; MOAA/S: Modified Observer’s Assessment of Alertness/Sedation Scale; i.v.: intravenous infusion; Std: standard stimuli; Dev: deviant stimuli; Nov: novel stimuli; ISI: interstimulus interval.

The AERP testing under propofol sedation was performed before the craniotomy surgery. Propofol was administered via a target-controlled infusion micro syringe pump (B. Braun, Germany). The sedation state was evaluated by the Modified Observer’s Assessment of Alertness/Sedation Scale (MOAA/S) separately by two experienced anesthesiologists, while the target concentration of propofol was increased gradually (0.2–0.5 μg/ml each time). The sedation state assessment was considered valid only when the two examiners’ results coincided. The study flow diagram is shown in Figure 1A.

*Awake state (A)*: The subjects were asked to remain awake while their eyes remained closed. Then, approximately 8 min of AERP data were collected.

*Light sedation (LS)*: The initial concentration was set at 0.5 μg/ml for target-controlled infusion of propofol, and the propofol concentration was gradually increased until the MOAA/S reached 3 (the subjects could only respond after his or her name was repeatedly and loudly called). After stabilization for 2 min, approximately 8 min of AERP data were collected.

*Deep sedation (DS)*: The propofol concentration was further increased until the MOAA/S score reached 1 (the subjects were unresponsive after mild tapping or shaking and only responded to painful stimuli). After stabilization for 2 min, AERP data were also collected.

*Recovery from anesthesia (R)*: Propofol infusion was ceased, and MOAA/S scores were assessed every 5 min until the subjects could respond correctly to the instructions. After the patients recovered consciousness, the AERP data were collected in the same manner. The patients were subsequently sent to the post-anaesthesia recovery room for further recovery.

During the intervention, if the anesthesiologists believed that the experiment needed to be stopped to ensure the safety of the subjects or if the subjects reported any uncomfortable symptoms, the experiment was terminated immediately.

### 2.4 Data acquisition and auditory stimuli based on the oddball paradigm

EEG data were recorded using 64 Ag-AgCl scalp electrodes placed according to the International 10–20 system (BrainAmp MR, Brain Products GmbH, Gilching, Germany). The impedances of the electrode remained below 20 kΩ, while most of the impedances were kept below 10 kΩ. The online reference electrode was CPz, with a sampling rate of 1,000 Hz.

The auditory stimulation paradigm included three kinds of acoustic stimuli: ① standard stimuli (Std, 500 Hz pure tone, duration 100 ms, *n* = 280, *p* = 70%), ② deviant stimuli (Dev, 1,000 Hz pure tone, duration 100 ms, *n* = 60, *p* = 15%) and ③ novel stimuli (Nov, patient’s own name generated by computer pronunciation, duration 1,000 ms, *n* = 60, *p* = 15%). There were 400 trials in each block, and the interstimulus interval between the presentation of each stimulus was between 800 and 1,200 ms. The total time of each block was approximately 8 min. Stimuli were presented pseudorandomly using E-Prime 3.0 software (Psychology Software Tools, Pittsburgh, PA). The deviant and novel stimuli were preceded by at least 2 continuous standard tones. The auditory stimulus sequences were presented through a pair of earphones (Sennheise, CX 80S), in which the volume was adjusted between 60 and 80 dB to ensure the comfort of the subjects. The auditory stimuli paradigm is shown in Figure 1B.

### 2.5 Electroencephalographic analysis

#### 2.5.1 Preprocessing

The raw EEG signals were processed using EEGLAB, a toolbox for MATLAB (MathWorks, Inc., Natick, MA). First, bandpass filters from 1 to 30 Hz were applied to the EEG data. EEG epochs synchronized with the onset of the auditory stimuli trial were extracted by a 1,500 ms time window (−500 ms-1,000 ms), and the prestimulus interval of each epoch was used to adjust the baseline.

Then, the EEG epochs were visually examined, and an independent component analysis (ICA) was used to correct trials involving eye blinks and body movements. After ICA, the EEG signals were re-referenced to the bilateral mastoid electrodes, and the sample size was decreased to 500 Hz.

#### 2.5.2 Time domain analysis

AERP waveforms for each participant elicited by auditory stimuli were categorized and averaged based on the auditory stimulus (standard, deviant, or novel) type. A group-level waveform was calculated by averaging the AERP waveforms of the participants, after which the scalp topographies were plotted according to the group-level waveform. We focused mainly on the MMN and P300 components. MMN and P300 waves were obtained by subtracting the averaged EEG signal of standard stimuli from the deviant/novel stimuli within 150–250 ms at the frontal (Fz) electrode site (Näätänen et al., 2007) and 300–500 ms at the central (Cz) electrode site (Polich, 2007), respectively. These epochs were baseline-corrected by −200 ms before the onset of auditory stimuli (Brockhoff et al., 2023; Demeter et al., 2022). The time windows and electrodes for measuring the amplitudes of the MMN and P300 were selected according to average AERP waveforms and scalp topographies at the group level and according to previous relevant oddball research.

#### 2.5.3 Time-frequency analysis

To identify theta-band oscillations relevant to the processing of different types of auditory stimulation, a short-time Fourier transform was applied to transform the time-domain information of the AERP response into the time-frequency domain. The spectrograms were corrected for each frequency using subtraction (reference interval: −400 to −100 ms corresponding to the beginning of the auditory stimulus). For each subject and stimulation type, we measured ERSP magnitudes in theta frequency bands. The theta-ERSP was obtained by averaging oscillation magnitudes at frontal (Fz, F3, F4, FC1, and FC2) electrodes in the 4–7 Hz frequency band (Javitt et al., 2018; Jiang et al., 2024) and within 200–500 ms after auditory stimulation onset.

### 2.6 Statistical analysis

AERP characteristics, including the average amplitudes of the MMN and P300, as well as the average power of the theta-ERSP oscillation, were obtained for each subject. The differences in these AERP characteristics between the MCI group and non-MCI group under different sedation conditions were assessed by Student’s t tests or Wilcoxon rank-sum tests as appropriate, while the auditory stimulus type was fixed. Spearman correlation was conducted to evaluate the relationships between the MoCA score and these AERP parameters. Multivariate linear regression was subsequently performed to control for age, education level, site of lesion, WHO pathological grade and combined symptomatic epilepsy as possible confounders.

Two-way repeated measures analysis of variance (ANOVA) was also conducted to compare the effects of *Sedation states* and neurocognitive *Groups* on AERP characteristics. The within-participant factor was different sedation state during propofol sedation (A vs. LS vs. DS vs. R), and the between-participant factor was group differentiated by neurocognitive performance (non-MCI group vs. MCI group). Bonferroni correction was applied for multiple comparisons.

Categorical data were compared using Pearson’s χ^2^ test and Fisher’s exact test. All the statistical analyses were implemented with IBM SPSS statistics 26 (IBM Corp., Armonk, NY). A two-tailed *p* value <0.05 was considered to indicate statistical significance.

## 3 Results

### 3.1 Demographics and characteristics

A total of 30 patients were enrolled in this study. One subject was excluded from the data analysis due to poor data quality (malfunction of left mastoid electrode). Twenty-nine eligible patients were included in the data analysis. Patients were divided into an MCI group (*n* = 13) and a non-MCI group (*n* = 16) according to the preoperative MoCA score. The patient clinical characteristics and demographic features are presented in Table 1.

**Table 1:** Clinical characteristics and demographic features of patients.

Variables	Total (*n* = 29)	Non-MCI group (*n* = 13)	MCI group (*n* = 16)	*P* value
Age, year	42.17 ± 10.25	39.31 ± 10.27	45.75 ± 7.48	0.071
Sex (male)	14 (48.3%)	6 (46.2%)	8 (50.0%)	0.837
BMI (kg m^−2^)	24.77 ± 3.38	24.91 ± 3.78	24.89 ± 3.13	0.984
Education level				0.362
College	16 (55.2%)	8 (61.5%)	8 (50.0%)	
Middle school	7 (24.1%)	4 (30.8%)	3 (18.8%)	
Primary school	6 (20.7%)	1 (7.7%)	5 (31.2%)	
ASA				0.114
I	19 (65.5%)	11 (84.6%)	8 (50.0%)	
II	10 (34.5%)	2 (15.4%)	8 (50.0%)	
MMSE	27.77 ± 1.93	28.45 ± 1.63	27.09 ± 2.02	0.097
MoCA	24.04 ± 3.84	27.00 ± 1.71	20.92 ± 3.23	<0.001*
HADS	11.00 ± 2.32	11.23 ± 2.80	10.63 ± 1.82	0.488
Propofol concentration (ug/ml)				
Light sedation (MOAA/S = 3)	1.82 ± 0.46	1.91 ± 0.44	1.75 ± 0.48	0.348
Deep sedation (MOAA/S = 1)	3.07 ± 0.63	3.25 ± 0.58	2.95 ± 0.65	0.212
Symptomatic epilepsy	11 (37.9%)	2 (15.4%)	9 (56.3%)	0.061
Site of lesion (left)	14 (48.3%)	5 (38.5%)	9 (56.3%)	0.340.
Main tumor location				0.451
Temporal and Insular	17 (58.6%)	9 (69.2%)	8 (50.0%)	
Frontal and Parietal	12 (41.4%)	4 (30.8%)	8 (50.0%)	
Midline shift	11 (37.9%)	4 (30.8%)	7 (43.8%)	0.702
WHO pathological grade				0.132
Grade1-2	21 (72.4%)	10 (76.9%)	11 (68.8%)	
Grade3-4	8 (27.6%)	3 (23.1%)	5 (31.2%)	

BMI: body mass index; ASA: American Society of Anesthesiology; MMSE: Mini-Mental State Examination; MoCA: Montreal Cognitive Assessment; HADS: Hospital Anxiety and Depression Scale; MOAA/S: Modified Observer’s Assessment of Alertness/Sedation Scale; WHO: World Health Organization.

### 3.2 AERP (MMN and P300) in MCI and non-MCI participants across different propofol-induced consciousness states

**Figure 2:**
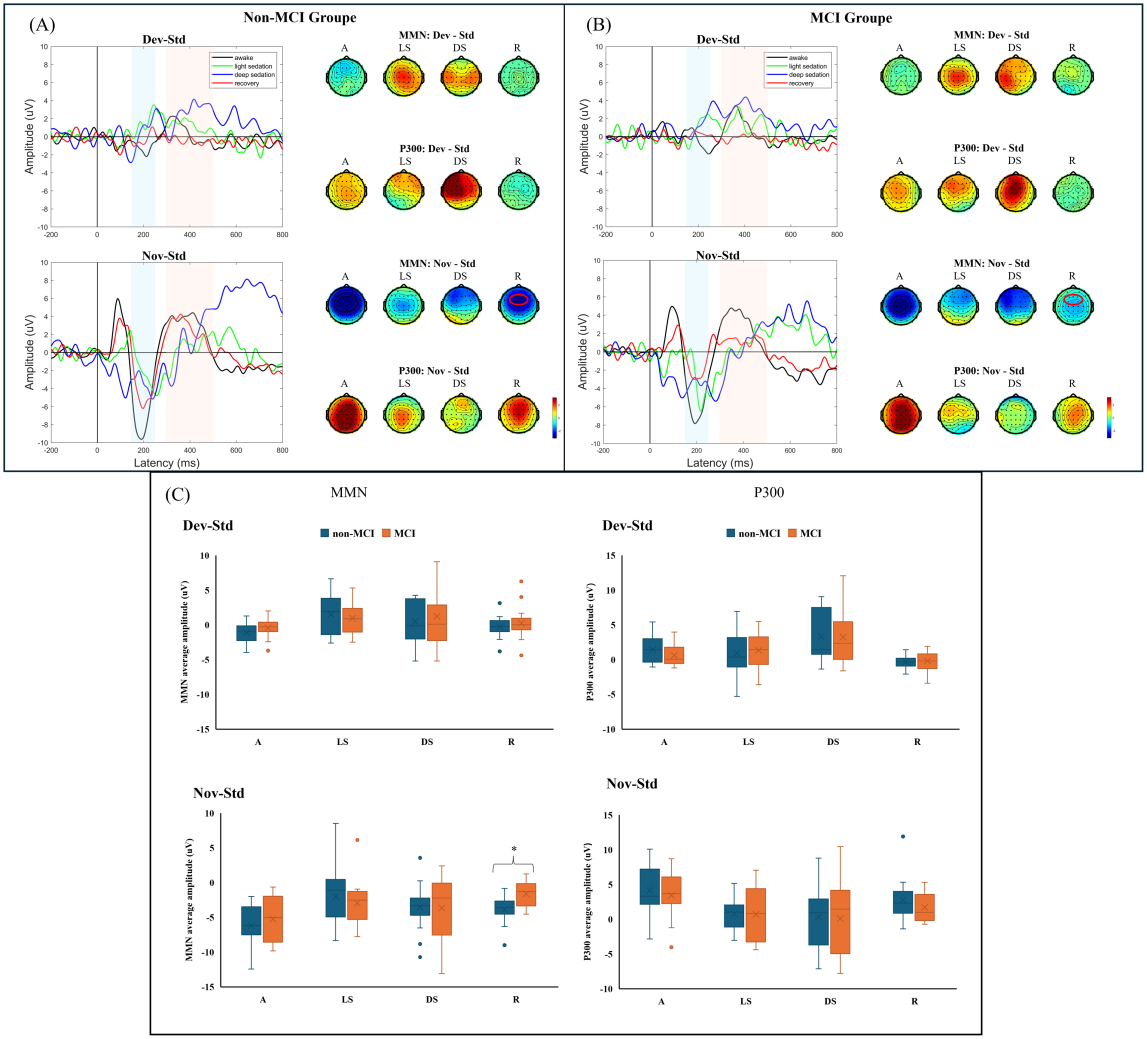
**(A)** AERP (MMN and P300) waveforms across different states of consciousness in the non-MCI group. **(B)** AERP (MMN and P300) waveforms across different states of consciousness in the MCI group. **(C)** Average MMN and P300 amplitudes between groups. MMN: mismatch negativity; AERP: auditory event-related potential; Std: standard stimuli; Dev: deviant stimuli; Nov: novel stimuli; A: awake; LS: light sedation; DS: deep sedation; R: recovery. *p < 0.05.

The obtained grand-average MMN and P300 waveforms from all participants at the Fz electrode during different states of propofol sedation are presented in Supplementary Figure S1. The average amplitudes of the MMN and P300 components in each group in the four sedation states were obtained from two difference waves (Dev-Std; Nov-Std) (Figures 2A,B). Compared to that in the non-MCI group, the average amplitude of the MMN component in the MCI group significantly decreased during recovery under the novel stimulus (−3.895 ± 1.961 μV vs. −1.617 ± 1.831 μV, *p* = 0.003). Moreover, there was no significant difference in the average MMN amplitude between the two groups during the other sedation states. Although the P300 amplitudes of the non-MCI group seemed greater than those of the MCI group under the novel stimuli, no significant difference was found in the P300 average amplitudes between the non-MCI and MCI groups under either deviation or novelty stimulation (Figure 2C). The exact values of the average AERP amplitude are shown in Supplementary Table S1.

### 3.3 Theta-ERSP oscillation between the MCI and non-MCI groups across different propofol-induced consciousness states

**Figure 3:**
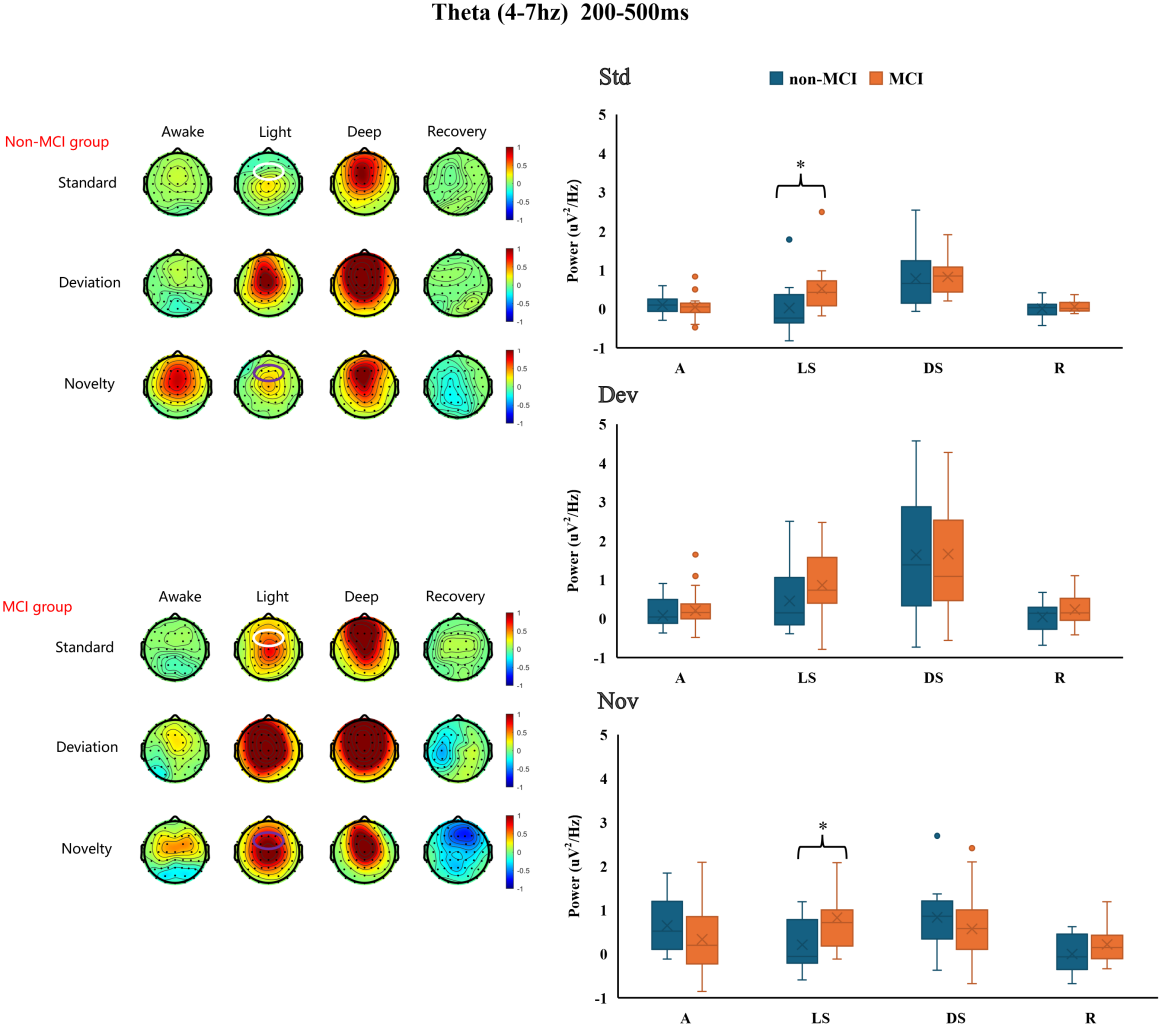
Differences in frontal theta-ERSP between the MCI group and the non-MCI group under different sedation conditions. Std: standard stimuli; Dev: deviant stimuli; Nov: novel stimuli; A: awake; LS: light sedation; DS: deep sedation; R: recovery. *p < 0.05.

The frontal theta-ERSP (4–7 Hz, 200–500 ms) signals from all participants during different states of propofol sedation are presented in Supplementary Figure S2. During light and deep sedation, the increase in frontal theta-ERSP in the MCI group was greater than that in the non-MCI group. These differences between the two groups were statistically significant for the standard (0.021 ± 0.658 μV^2^/Hz vs. 0.515 ± 0.622 μV^2^/Hz, *p* = 0.048) and novel (0. 212 ± 0.584 μV^2^/Hz vs. 0.823 ± 0.931 μV^2^/Hz, *p* = 0.041) under light sedation (Figure 3). The exact values of theta-ERSP power are shown in Supplementary Table S1.

### 3.4 The effects of sedation states and preoperative neurocognitive performance on AERP characteristics

The results of two-way repeated measures ANOVA showed that *Sedation states* revealed significant main effects on all the AERP characteristics, including the average amplitudes of the MMN and P300 as well as the average power of the theta-ERSP oscillation, while there was no significant main effect between *Groups* under different stimulation types. Additionally, the two-way interaction of *Sedation states* and *Groups* was not significant either. Relevant statistics are summarized in Supplementary Table S2.

### 3.5 The relationship between preoperative cognitive performance (MoCA scores) and neural response across different propofol-induced consciousness states

As shown in Table 2, there were correlations between preoperative MoCA scores and several AERP parameters. The average MMN amplitude induced by deviation stimuli in the awake state was inversely related to preoperative cognitive performance (*r* = −0.480, *p* = 0.013). During the recovery period, the MMN amplitude induced by novel stimuli was also negatively correlated with the preoperative MoCA score (*r* = −0.391, *p* = 0.036). A positive increase in the average MMN amplitude might be associated with cognitive impairment. An increase in the theta-ERSP during light sedation was associated with a decrease in the preoperative MoCA score under all standard (*r* = −0.490, *p* = 0.011), deviation (*r* = −0.434, *p* = 0.027), and novel (*r* = −0.415, *p* = 0.035) stimulation conditions (the linear correlation is displayed in Supplementary Figure S3). After adjusting for age, education level, site of lesion, WHO pathological grade and combined with symptomatic epilepsy by multivariable linear regression, the correlations between the average MMN amplitude induced by novel stimuli and preoperative MoCA score during the recovery period remained significant (*β* = −0.356, *p* = 0.035), as well as the standard (*β* = −0.491, *p* = 0.011) and novel (*β* = −0.594, *p* = 0.007) stimuli during light sedation of frontal theta-ERSP. Therefore, compared to those with intact cognition, patients with preoperative cognitive impairment might demonstrate more intense theta-ERSP increases during the auditory stimulation task under light sedation (Table 3).

**Table 2:** Relationship between preoperative MoCA scores and AERP parameters.

Spearman correlation	MMN amp, Fz				P300 amp, Cz				theta-ERSP					
Sedation stated · MoCA score	Dev-Std		Nov-Std		Dev-Std		Nov-Std		Std		Dev		Nov	
	*r*	*p*	*r*	*p*	*r*	*p*	*r*	*p*	*r*	*p*	*r*	*p*	*r*	*p*
A	−0.480	0.013*	−0.094	0.647	0.279	0.150	0.139	0.499	0.076	0.712	−0.189	0.356	0.066	0.749
LS	−0.075	0.716	0.035	0.864	−0.148	0.470	0.048	0.817	−0.490	0.011*	−0.434	0.027*	−0.415	0.035*
DS	0.042	0.838	0.037	0.858	−0.102	0.622	0.076	0.711	−0.010	0.963	0.038	0.854	0.017	0.933
R	0.081	0.695	−0.391	0.036*	−0.231	0.267	0.013	0.950	0.013	0.949	0.218	0.284	0.109	0.597

MMN amp: the average amplitude of mismatch negativity; P300 amp: the average amplitude of P300. Std: standard stimuli; Dev: deviant stimuli; Nov: novel stimuli; A: awake; LS: light sedation; DS: deep sedation; R: recovery; ERSP: event-related spectral perturbation; MoCA: Montreal Cognitive Assessment Scale; r: Spearman correlation coefficient; *p < 0.05.

**Table 3:** Relationships between preoperative MoCA scores and AERP parameters corrected by multivariable linear regression.

Multivariable liner regression	MMN amp, Fz				P300 amp, Cz				theta-ERSP					
Sedation stated · MoCA score	Dev-Std		Nov-Std		Dev-Std		Nov-Std		Std		Dev		Nov	
	*β*	*p*	*β*	*p*	*β*	*p*	*β*	*p*	*β*	*p*	*β*	*p*	*β*	*p*
A	−0.440	0.075	−0.133	0.482	0.161	0.436	0.257	0.374	0.090	0.742	0.161	0.561	0.222	0.449
LS	−0.220	0.476	0.234	0.458	0.256	0.388	0.165	0.557	−0.491	0.011*	−0.424	0.057	−0.594	0.007*
DS	−0.342	0.149	0.341	0.242	0.103	0.719	0.086	0.736	−0.369	0.164	−0.287	0.237	−0.269	0.151
R	0.119	0.583	−0.356	0.035*	−0.109	0.678	0.270	0.394	−0.254	0.361	0.116	0.551	0.207	0.358

The covariates including age, education level, site of lesion, WHO pathological grade and combined with symptomatic epilepsy were corrected by multiple linear regression. MMN amp: the average amplitude of mismatch negativity; P300 amp: the average amplitude of P300. Std: standard stimuli; Dev: deviant stimuli; Nov: novel stimuli; A: awake; LS: light sedation; DS: deep sedation; R: recovery; ERSP: event-related spectral perturbation; MoCA: Montreal Cognitive Assessment Scale; β: standardization coefficient; *p < 0.05.

## 4 Discussion

In this study, we explored and compared the characteristics of AERPs, including the MMN, P300 and theta-ERSP, in patients with different propofol-induced consciousness states between the non-MCI group and MCI group. Differences in certain neural responses were found in the auditory information processing of the brain between groups. Furthermore, we found that frontal theta-ERSP induced by standard and novel stimuli in the light sedation state was inversely related to preoperative MoCA scores, and the same trend was also observed for the average MMN amplitude induced by novel stimuli in the recovery period.

Given that the AERPs measure the spatial information processing rate at large neural networks, they are applied for the assessment of cognitive impairment, especially in the neurodegenerative and neuropsychiatric disorders with associated cognitive deficits (Olichney et al., 2022). The oddball paradigm is the most common paradigm used to analyze cognitive processes and detect neurocognitive changes (Ferrari et al., 2010). During the oddball paradigm processing, the infrequent stimuli are distinguished from regular stimuli and elicit corresponding neural response components, with MMN and P300 being the most widely examined AERP components. Generally, MMN represents the automatic process of deviance detection, which reflects the brain’s automatic detection capability to irregular input (Garrido et al., 2009). P300 is the most notable late- latency AERP component, which is frequently analyzed in cognitive research and associated with variety of cognitive processes, such as attention allocation, working memory updating, perceptual discrimination, etc. (Duncan et al., 2009).

ERP components have been considered indicators of cognitive decline in previous studies (Papaliagkas et al., 2011). MMN has become a prognostic feature of MCI progression (Morrison et al., 2018). The P300 component, on the other hand, has been shown to have significant differences in latency and amplitude between controls and MCI patients (Medvidovic et al., 2013; Gu and Zhang, 2017). However, other comparable studies have revealed only slight differences in P300 features (Lee et al., 2013), and there is still controversy regarding the ERP characteristics of patients with MCI. In this study, we found that the average amplitude of the MMN component in the MCI group significantly decreased during the recovery period after the novel stimulus, while no significant differences were observed in the P300 amplitude. Heterogeneity in the auditory task paradigm design may account for inconsistent results in similar studies.

Visual, auditory, and tactile stimuli are the major sensations that evoke ERPs. Auditory information is often input by prior knowledge learning and is processed at the cognitive level after a meaningful or preset auditory event. There are different response patterns for pure tone stimuli and semantic sounds with meaning during cognitive processes (Tsolaki et al., 2017). Similar results were observed in our study. We used an oddball paradigm containing 3 stimulation types, where the novel stimulus was a synthesis of the pronunciation of the subject’s name. We found that the ERP amplitude evoked by the deviant stimulus, which was also a pure tone, was smaller than that evoked by the novel stimulus in each sedation state. This may be because the brain has different processing patterns for pure tone and semantic stimuli, and familiar semantic information could attract more attention as well as cognitive resources (Park et al., 2023).

Notably, a slowing of the EEG rhythm has been observed in patients with Alzheimer’s disease-related CI (Lorek et al., 2023). Comparing CI patients to controls, it has been discovered that theta band power increases while alpha band power decreases (Chu et al., 2023). Increased delta and theta activity could distinguish between individuals with MCI and those with pathological cognitive declines (Good, 2003). Moreover, previously study has showed that the ERSP responses under the task state could discriminate healthy elderly from MCI and Alzheimer’s disease patients (Fraga et al., 2018). Thus, the regulation of neuronal oscillations might provide a causal relationship between neural activities and cognitive processes, offering essential insights into human brain functionality (Herrmann et al., 2016).

Theta oscillations are associated with working memory updating, conflict monitoring and multiple other cognitive control processes (Cavanagh and Frank, 2014; Sauseng et al., 2010) as well as functional inhibition facilitating executive function (Huster et al., 2013). A study examined theta oscillations in MCI patients during a semantic go/no-go task. The results indicated that greater theta oscillation synchronization was associated with a greater degree of CI on Go trials at central electrodes (Lydon et al., 2022). In the task state, the activity in the theta band was related to the participation of cognitive resources in the brain. When performing the same task, cerebral cortex with impaired cognitive function tend to allocate more cognitive resources (Puma et al., 2018). Therefore, neural activity in the task state may provide more reliable information about subtle changes in cortical network function than in the resting state (Deiber et al., 2015).

Anesthetics and sedatives can also modulate EEG oscillations. With increasing concentrations of propofol, EEG activity is gradually dominated by a lower frequency oscillation when patients are in a state of deep sedation (Purdon et al., 2013). Pertinently, the emergence of a stable slow wave oscillation in the theta band may indicate loss of consciousness (Huang et al., 2018). In the present study, we observed a significant difference in the frontal theta ERSP between the non-MCI and MCI groups. Although there is not adequate evidence to interpret these task-related changes during sedation, our findings indicate that CI may affect auditory information processing and that the administration of propofol and changes in consciousness further exacerbate these preexisting deficits in neurosurgical patients.

Several studies have reported that perioperative anesthesia management (e.g., anesthetic depth) and brain function monitoring via processed electroencephalography can reduce the risk of postoperative CI (Evered et al., 2021; Sumner et al., 2023). Neurosurgery and preoperative CI are independent risk factors for the development of perioperative neurocognitive disorders (Wang et al., 2020; Huang et al., 2022), and anesthetics and sedatives have provided a unique opportunity to conduct a “stress test” for these vulnerable brains (Berger et al., 2023). When administering general anesthetics to patients with pathological brain states, the importance of brain function monitoring is greater than ever. Our results suggested that the AERP can be used to evaluate the brain state in real time throughout the perioperative period. Therefore, perioperative AERP might be a feasible method for identifying electrophysiological markers indicative of reduced brain integrity and cognitive decline.

There were also methodological limitations in this study. First, some subjects in our study who had combined symptomatic epilepsy were taking valproic acid as an antiepileptic drug. Previous study has indicated that the use of antiepileptic drugs was associated with posterior alpha rhythm slowing as well as generalized intermittent slow waves, but valproic acid was not associated with either of these outcomes (Limotai et al., 2020). Besides, none of the subjects had seizures within the most recent month before the AERP testing. Second, we did not limit the location of gliomas to a particular site. Although the site of the tumor was not significantly different between the groups, this difference might increase the heterogeneity among subjects and limit the external validity of our results. We further applied multivariable liner regression to correct for confounders, including age, education level, site of lesion, WHO pathological grade and combined with symptomatic epilepsy. In addition, we focused only on task-related oscillations in the frontal theta band under different sedation conditions, but the impact of preoperative cognitive deterioration may have affected the global brain scale score. Finally, the effect of MCI grouping on AERP characteristics did not reveal significant main effects in the two-way repeated measure ANOVA analysis, which we speculate may be due to the limitation of sample size and the heterogeneity among the data. In general, investigations of AERP characteristics could improve the understanding of perioperative neuro-electrophysiological markers associated with cognitive decline. We believe that perioperative MMN and theta-ERSP might be promising “predictors” of perioperative cognitive decline in neurosurgical patients.

## 5 Conclusion

Compared to glioma patients with intact cognitive function, those with MCI demonstrated significant differences in MMN and theta oscillations under different propofol-induced consciousness states. Moreover, after adjusting After adjusting for age, education level, site of lesion, WHO pathological grade and combined with symptomatic epilepsy by multivariable linear regression, the frontal theta-ERSP induced by standard and novel stimuli in the light sedation state was inversely related to the preoperative MoCA score, and the same trend was also observed for the average MMN amplitude induced by novel stimuli during the recovery period.

## Data Availability

The raw data supporting the conclusions of this article will be made available upon reasonable request from the corresponding author.
