# Supplementary material for: The characteristics of auditorial event-related potential under propofol sedation associated with preoperative cognitive performance in glioma patients
Source: Front Neurosci. 2024 Nov 14;18:1431406. doi: 10.3389/fnins.2024.1431406 (PMC11603416; doi:10.3389/fnins.2024.1431406)

Supplementary Material

**Supplementary Figure 1.** The auditory event-related potentials across different states of consciousness. The grand average (29 subjects) AERP waves in each sedation state were extracted from the Fz electrode. Std: standard stimuli; Dev: deviant stimuli; Nov: novel stimuli; diff 1: Dev-Std difference wave; diff 2: Nov-Std difference wave.


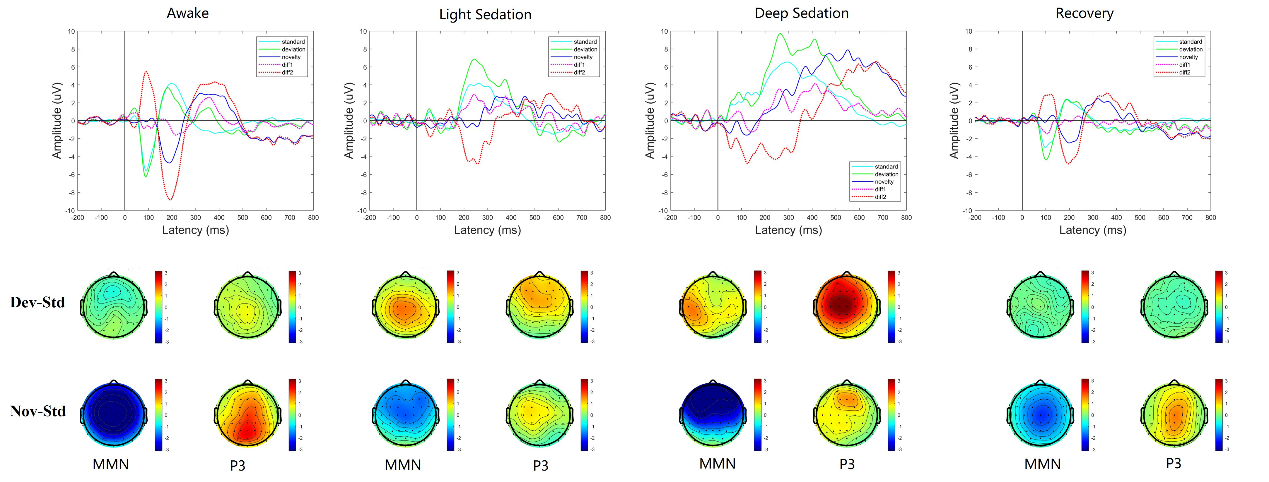

Supplement: Supplementary file 1 [file Data_Sheet_1.docx]
