# Supplementary material for: The characteristics of auditorial event-related potential under propofol sedation associated with preoperative cognitive performance in glioma patients
Source: Front Neurosci. 2024 Nov 14;18:1431406. doi: 10.3389/fnins.2024.1431406 (PMC11603416; doi:10.3389/fnins.2024.1431406)

Supplementary Material

**Supplementary Figure 2.** The theta ERSP was also obtained at frontal (Fz, F3, F4, FC1, FC2) electrode sites by averaging the oscillation magnitudes in the 4-7 Hz frequency range and within 200–500 ms after auditory stimulation onset. *:P<0.05; **P<0.01


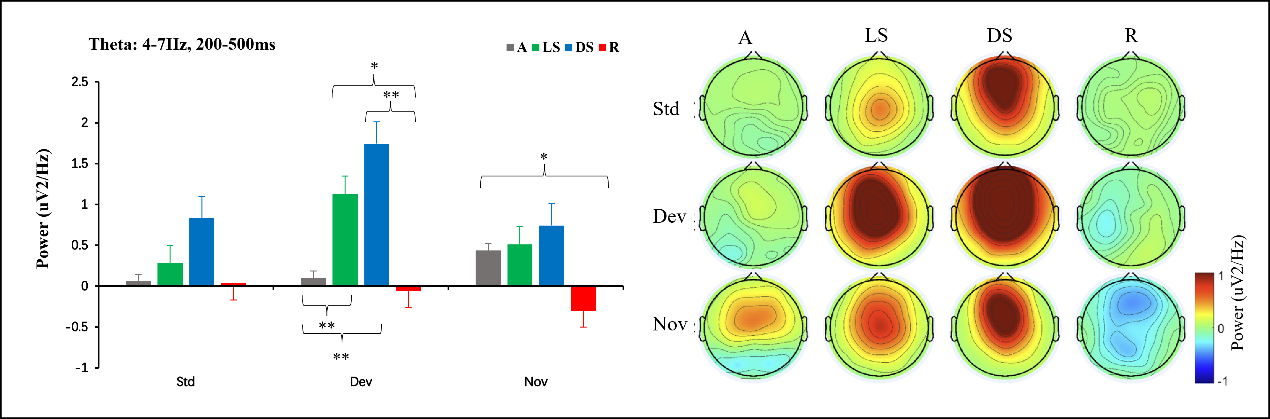

Supplement: Supplementary file 2 [file Data_Sheet_2.docx]
