# Supplementary material for: The characteristics of auditorial event-related potential under propofol sedation associated with preoperative cognitive performance in glioma patients
Source: Front Neurosci. 2024 Nov 14;18:1431406. doi: 10.3389/fnins.2024.1431406 (PMC11603416; doi:10.3389/fnins.2024.1431406)

Supplementary Material

**Supplementary Figure 3.** Associations between AERP parameters and preoperative MoCA scores Std: standard stimuli; Dev: deviant stimuli; Nov: novel stimuli; A: awake; LS: light sedation; DS: deep sedation; R: recovery; ERSP: event-related spectral perturbation; MoCA: Montreal Cognitive Assessment Scale; r: Spearman correlation coefficient


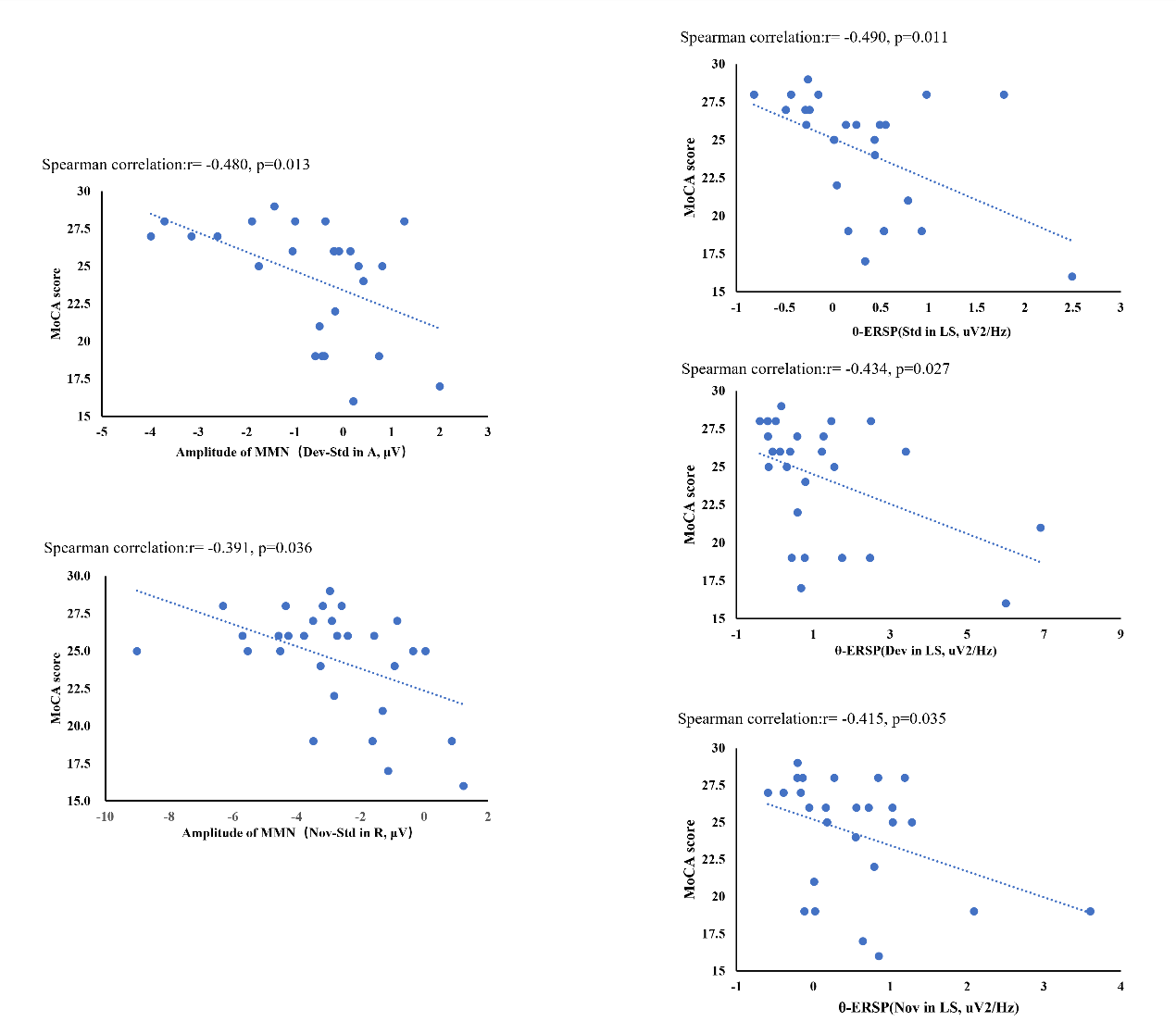

Supplement: Supplementary file 3 [file Data_Sheet_3.docx]
