# Supplementary material for: The characteristics of auditorial event-related potential under propofol sedation associated with preoperative cognitive performance in glioma patients
Source: Front Neurosci. 2024 Nov 14;18:1431406. doi: 10.3389/fnins.2024.1431406 (PMC11603416; doi:10.3389/fnins.2024.1431406)
Supplement: Supplementary file 4 [file Table_1.docx]

Supplementary Material

# Supplementary Tables

For more information on Supplementary Material and for details on the different file types accepted, please see [here](https://www.frontiersin.org/guidelines/author-guidelines#supplementary-material).

Supplementary Table 1. Characteristics of the MMN, P300 and theta-ESRP between the non-MCI group and MCI group.

|  | **Non-MCI group (n=13)** | **MCI group (n=16)** | *P* |
| --- | --- | --- | --- |
| **Average amplitude of MMN(μV)** |  |  |  |
| **Dev-Std** |  |  |  |
| A | -1.117±1.470 | -0.420±1.355 | 0.196 |
| LS | 1.491±2.947 | 0.947±2.285 | 0.579 |
| DS | 0.455±3.089 | 1.235±5.669 | 0.660 |
| R | -0.252±1.644 | 0.244±2.395 | 0.531 |
| **Nov-Std** |  |  |  |
| A | -6.223±3.092 | -5.246±3.191 | 0.411 |
| LS | -1.991±4.237 | -2.956±3.557 | 0.519 |
| DS | -3.538±3.363 | -3.666±4.588 | 0.934 |
| R | -3.895±1.961 | -1.617±1.831 | 0.003* |
| **Average amplitude of P300(μV)** |  |  |  |
| **Dev-Std** |  |  |  |
| A | 1.242±1.966 | 0.616±1.606 | 0.233 |
| LS | 0.938±3.19 | 1.337±2.365 | 0.702 |
| DS | 3.323±3.6 | 3.233±4.177 | 0.951 |
| R | -0.311±0.937 | -0.186±1.48 | 0.800 |
| **Nov-Std** |  |  |  |
| A | 4.133±3.502 | 3.414±3.368 | 0.581 |
| LS | 0.754±2.147 | 0.691±3.971 | 0.958 |
| DS | 0.311±4.533 | 0.103±5.292 | 0.910 |
| R | 2.784±3.055 | 1.695±2.093 | 0.299 |
| **Power of theta-ERSP (μV^2^ Hz^-1^)** |  |  |  |
| **Std** |  |  |  |
| A | 0.103±0.242 | 0.047±0.321 | 0.607 |
| LS | 0.021±0.658 | 0.515±0.622 | 0.048* |
| DS | 0.780±0.799 | 0.821±0.491 | 0.870 |
| R | 0.018±0.223 | 0.057±0.15 | 0.469 |
| **Dev** |  |  |  |
| A | 0.077±0.571 | 0.203±0.659 | 0.592 |
| LS | 0.452±0.833 | 0.860±0.849 | 0.230 |
| DS | 1.641±1.545 | 1.657±1.494 | 0.979 |
| R | 0.041±0.393 | 0.231±0.371 | 0.200 |
| **Nov** |  |  |  |
| A | 0.647±0.651 | 0.335±0.742 | 0.244 |
| LS | 0.212±0.584 | 0.823±0.931 | 0.041* |
| DS | 0.833±0.765 | 0.572±0.873 | 0.411 |
| R | -0.014±0.460 | 0.218±0.446 | 0.231 |

MCI: mild cognitive impairment; MMN: mismatch negativity; Std: standard stimuli; Dev: deviant stimuli; Nov: novel stimuli; A: awake; LS: light sedation; DS: deep sedation; R: recovery; ERSP: event-related spectral perturbation; *: p<0.05

Supplementary Table 2. Two-way repeated measures ANOVA of 4 Sedation states ((A vs. LS vs. DS vs. R) * 2 Groups (non-MCI vs MCI) in MMN, P300 and theta-ERSP

| **Two-way repeated measures ANOVA** | *F* **^b^** | *P* | *η^2^_p_* |
| --- | --- | --- | --- |
| **MMN amp (Dev-Std）** |  |  |  |
| Sedation states | 4.544 | 0.011* | 0.353 |
| Groups | 0.431 | 0.517 | 0.016 |
| Sedation states * Groups | 0.426 | 0.736 | 0.049 |
|  |  |  |  |
| **MMN amp (Nov-Std)** |  |  |  |
| Sedation states | 8.473 | 0.000 * | 0.504 |
| Groups | 1.409 | 0.251 | 0.050 |
| Sedation states * Groups | 1.993 | 0.141 | 0.193 |
|  |  |  |  |
| **P300 amp (Dev-Std）** |  |  |  |
| Sedation states | 9.109 | 0.000 * | 0.532 |
| Groups | 0.235 | 0.632 | 0.009 |
| Sedation states * Groups | 0.379 | 0.769 | 0.045 |
|  |  |  |  |
| **P300 amp (Nov-Std)** |  |  |  |
| Sedation states | 5.746 | 0.004 * | 0.418 |
| Groups | 0.791 | 0.382 | 0.030 |
| Sedation states * Groups | 0.028 | 0.993 | 0.004 |
|  |  |  |  |
| **Theta-ERSP (Std)** |  |  |  |
| Sedation states | 3.442 | 0.032 * | 0.292 |
| Groups | 2.456 | 0.092 | 0.057 |
| Sedation states * Groups | 2.302 | 0.102 | 0.216 |
|  |  |  |  |
| **Theta- ERSP (Dev)** |  |  |  |
| Sedation states | 5.172 | 0.006 * | 0.383 |
| Groups | 2.162 | 0.153 | 0.074 |
| Sedation states * Groups | 0.593 | 0.448 | 0.021 |
|  |  |  |  |
| **Theta- ERSP (Nov)** |  |  |  |
| Sedation states | 3.671 | 0.016 * | 0.120 |
| Groups | 2.027 | 0.063 | 0.048 |
| Sedation states * Groups | 2.260 | 0.088 | 0.079 |

ANOVA: analysis of variance; Std: standard stimuli; Dev: deviant stimuli; Nov: novelty stimuli; amp: average amplitude; ERSP: event-related spectral perturbation; *F* **^b^** : Bonferroni correction was applied for multiple comparisons *: p<0.05.
